# Gastric adenocarcinoma: 1-year overall survival, disability-adjusted life years, years of life lost, and prognostic factors—a single-institution experience

**DOI:** 10.3389/fonc.2022.918833

**Published:** 2022-09-08

**Authors:** Tatiane Tiengo, Gisele Aparecida Fernandes, Maria Paula Curado

**Affiliations:** ^1^ Post Graduation Program A.C. Camargo Cancer Center, São Paulo, Brazil; ^2^ Nucleus of Epidemiology and Statistics in Cancer, A. C. Camargo Cancer Center, São Paulo, Brazil

**Keywords:** gastric adenocarcinoma, survival, prognostic factor, years of life lived with disability, years of life lost

## Abstract

**Objective:**

To analyze factors affecting 1-year overall survival and burden of gastric adenocarcinoma in a single-institution cohort.

**Methods:**

A prospective cohort study of gastric adenocarcinoma patients from a cancer center in São Paulo, Brazil, was conducted between February 2016 and July 2019. Overall survival was analyzed at 12 months post-diagnosis using the Kaplan–Meier method. A log-rank test was applied to compare curves. Sociodemographic and clinicopathological features were assessed to detect prognostic factors using univariate and multivariable Cox regression analyses to calculate hazard ratio (HR) and its confidence intervals (CIs). Disability-adjusted life years (DALY) constituted the sum of years of life lost (YLL) plus years lived with disability (YLD). YLL represented the sum of years lost before the age of 76.6 years. YLD was calculated as the number of cases multiplied by the duration and burden of the disease. YLL per death was calculated as the mean YLL for each individual.

**Results:**

Overall survival at 1-year follow-up was 80.8%. The multivariable model adjusted for age and sex identified cerebrovascular disease (HR 8.5, 95% CI 3.3–21.8), stage III/IV (HR 5.7, 95% CI 2.3–13.7), diabetes (HR 3.2, 95% CI 1.5–6.6), and<9 years of education (HR 2.9, 95% CI 1.5–5.8) as prognostic factors. Out of the 214 treated cases, there was 700.72 DALY during the first year, of which 90.55% corresponded to YLL and 9.45% to YLD. The average YLL per death was 15.48 and was higher among women (19.24 YLL per death).

**Conclusion:**

At a single cancer center, 1-year overall survival probability was approximately 80% in patients with gastric adenocarcinoma. Patients with a higher risk of death had cerebrovascular disease, advanced clinical staging, diabetes, and/or lower educational level. Approximately 700 years of DALY was documented, with women having the highest YLL per death. Because this study was conducted at a single cancer center, the results might not be representative of a general population. To the best of our knowledge, this study was the first to assess gastric adenocarcinoma DALY, YLL, and YLL per death in the first year of follow-up in a hospital cohort in Brazil.

## Introduction

In 2020, there were approximately 1.1 million new cases of gastric cancer and 769,000 related deaths globally (1). In Brazil, gastric cancer is the fourth and sixth most common type in men (13,360 new cases) and women (7,870 new cases), respectively. In 2019, gastric cancer was responsible for 7.9% of cancer deaths in men and 5% in women (2). Most cases of gastric cancer are adenocarcinomas (3, 4); the main risk factors of gastric adenocarcinoma are age >60 years (3, 5), male gender (5–7), low socioeconomic status, occupational exposure (high temperatures and dust, asbestos, X and gamma radiation, inhalation of hexavalent chromium, and inorganic lead compounds) (8), smoking, alcohol consumption, obesity, gastroesophageal reflux disease, *Helicobacter pylori* infection, high consumption of salt and nitrites, and low consumption of fresh fruit and vegetables (3, 4, 9). Prognostic factors include the presence of comorbidities (10, 11), smoking, alcohol consumption (12), clinical stage (13–15), and clinical and molecular characteristics of the tumors (15–17).

Gastric cancer survival remains low in most countries, with 5-year survival (2010–2014) rates of 20%–40% (18) and 1-year survival rates of 59.7% in the United States (19) and 57% in Denmark (20). Survival probability is even lower when based on the clinical stage. In the United States, for cases diagnosed at clinical stage I, overall survival is 80.6% in the first year, 64.9% at 3 years, and 56.7% at 5 years (15). For clinical stage IV cases, overall survival was 28.3% in the first year, 7.8% after 3 years, and 5.0% after 5 years (15). A single-institution experience in Brazil has overall survival, ranging from 87.3% to 76.5% in the first year of follow-up for early tumors (stages I and II) and falling to 27.2% in metastatic cases (stage IV) (21).

Gastric cancer has a major social and economic impact. In 2017, it accounted for 19.1 million disability-adjusted life years (DALY), of which 98% was due to years of life lost (YLL) and 2% was due to years lived with disability (YLD). This disease represents the third leading cause of DALY for cancer in men (5). In Brazil, the Global Burden of Disease Study estimated that gastric cancer was responsible for 261.54 DALY per 100,000 population, compared with 323.29 DALY per 100,000 in Colombia, 472.18 DALY per 100,000 in Chile, 107.75 DALY per 100,000 in South Africa, 118.23 DALY per 100,000 in the United States, and 165.60 DALY per 100,000 in Denmark (22).

In general, survival studies focus on elucidating the severity of disease and prognostic factors, whereas pooled analyses of DALY, YLL, YLD, and YLL per death provide information on the burden of disease. Analyzing these indicators together in a hospital cohort provides key insights into the burden and severity of gastric adenocarcinoma in populations with access to healthcare. This is important, as few studies have investigated this issue in Brazil. The main purpose of this study was to estimate overall survival from gastric adenocarcinoma at 1-year follow-up, along with prognostic factors. Within this framework, DALY, YLL, and YLL per death were evaluated in patients with gastric adenocarcinoma.

## Materials and methods

### Data source

This is a prospective cohort of cases in São Paulo (Brazil), which forms part of a larger multicenter case–control study. This study included 214 patients recruited from A. C. Camargo Cancer Center in São Paulo, Brazil, between February 2016 and July 2019. São Paulo is the largest city in Brazil and is one of the most populous areas in Latin America, with 12,396,372 inhabitants, and it had a human development index of 0.805 in 2010 (23). The study data were drawn from the module “Epidemiology of Gastric Adenocarcinomas in Brazil”, which is part of the project “Epidemiology and Genomics of Gastric Adenocarcinomas in Brazil”. A. C. Camargo Cancer Center was founded 69 years ago and is a referral hospital for cancer treatment in Brazil and South America, serving patients from both public and private or health insurance. The cancer center runs a post-doctoral training program and oncologic training in surgery, radiotherapy, chemotherapy, and clinical trials. The center also conducts translational research to support cancer patients (24).

### Patients

Cases were interviewed by a trained nurse or nutritionist. All patients completed an epidemiological questionnaire to collect sociodemographic, lifestyle, and clinical information. Study data were stored on the REDCap platform hosted by the A. C. Camargo Cancer Center (25, 26). The inclusion criteria were cases with gastric adenocarcinoma (ICD O3 C16, M-8140/3) (27) confirmed by histology without previous cancer diagnoses, except for non-melanoma skin cancer and patients aged 18–75 years. The exclusion criterion was patients with physical or psychological limitations in answering the questionnaire.

Vital status was recorded through passive and active follow-up. Passive follow-up involved using the most recent information available from medical records (day, month, and year). Active follow-up was obtained from the Regional Electoral Court from the death certification registry held by the state and federal governments for all patients. Death was coded as death from cancer, death by other causes, or lost to follow-up when the sources searched yielded no information. There were no losses in the first year of follow-up in this study.

### Variables

The assessed variables were gender (female and male), ethnicity (white and non-white, with the latter being composed of black, brown, and yellow ethnicity), education (grouped into<9 years corresponding to illiterate and elementary and >10 years—high school and university), access to the public health service (Brazilian national health service—SUS) or private or health insurance, tumor location (cardia or non-cardia), and clinical stage (categorized into three groups: I/II, III, and IV). These three stage groupings were used to analyze the YLL due to death and were grouped into I/II and III/IV in the survival analysis.

The parameters assessed in the survival analysis were age (<60 and ≥60 years), marital status (married or unmarried, with the latter including single, widowed, and divorced), tobacco consumption (smokers/ex-smokers and non-smokers), alcohol consumption (no or yes), body mass index (BMI) (classified as underweight [<18.5 kg/m^2^], normal weight [18.5–24.99 kg/m^2^], overweight [25–29.99 kg/m^2^], and obese [>30 kg/m^2^]) (28), *Helicobacter pylori* infection (negative or positive), Lauren’s histological type (intestinal, diffuse, mixed, or not otherwise specified (NOS) adenocarcinoma) (29), histological grade (well differentiated, moderately differentiated, poorly differentiated, undifferentiated, or information not available), human epidermal growth factor receptor 2 (HER2) (negative for results 0 and 1+, doubtful for 2+, and positive for 3+) (30), treatment (yes or no), and presence of comorbidities before cancer diagnosis (using the Charlson Comorbidity Index [CCI]). This index was grouped into three categories based on scores (0, 1 or 2, and ≥3), where the criteria “any tumor” (2 points) and “metastatic solid tumor” (6 points) were not included in the sum (31).

### Statistical analysis

Descriptive data analysis was performed using absolute and relative frequencies. For group comparisons, Pearson’s chi-square test or Fisher’s exact test was used, as appropriate.

Overall survival was defined as the interval in months between the date of diagnosis and the date of final information or death from any cause. The Kaplan–Meier product-limit estimator and log-rank test were applied to evaluate curves. Cox semiparametric proportional hazard model was used to assess the prognostic potential of each variable and to calculate hazard ratio (HR) and its 95% confidence interval (95% CI). The proportional hazards assumption was based on Schoenfeld residuals. Variables with p< 0.20 were selected for multivariable analysis, in which those with p ≤ 0.05 and those that contributed to the goodness of fit of the model were retained. For epidemiological criteria, age and sex were retained to adjust the model.

Disability-adjusted life years (DALY) is a summary measure that combines YLL and YLD (32, 33). YLL is the sum of years of life lost for each individual. It is obtained by subtracting the age at death from life expectancy and was used for 2019 in the state of São Paulo (76.6 years) (34) with the following formula (1) (33, 35):


(1) $$YLL\;=\;\sum_{i=0}^{76.6}\left(i\right)\times \left(76.6-i\right)$$

The calculation of YLD was performed by multiplying the number of cases (n) by the mean duration of the disease (t) and the weight factor that reflects the severity of the disease (pd), with the following formula (2) (32, 33, 35):


(2) YLD=n ×t ×pd

Disease duration for individuals who survived the first year of follow-up corresponded to 1 year (study period), and the burden of the disease was 0.288 for cases of localized gastric adenocarcinoma at diagnosis and 0.451 for metastatic cases (32, 33).

Years of life lost per individual (YLL per death) was calculated by dividing YLL by the total number of deaths for each variable. Survival analyses, as well as graphs and images, were implemented in RStudio version 1.3.959. DALY, YLL, and YLD calculations were performed using the statistical program SPSS version 22.0. Excel was used to construct graphs.

## Results

**Figure 1:**
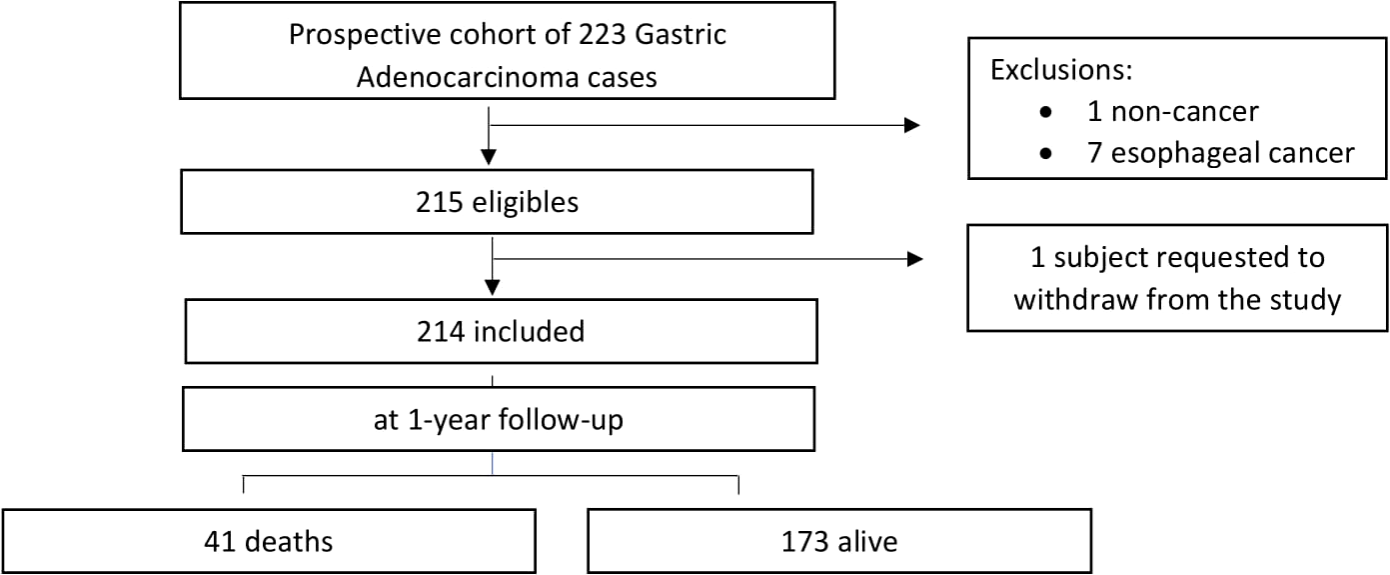
Summary of Gastric Adenocarcinoma cases status after one year follow-up and flowchart of exclusion criteria.

Of the initial 223 patients, nine were excluded. Of the nine excluded cases, one had no confirmation of adenocarcinoma in the histological analyses, seven had esophageal adenocarcinoma, and one asked to withdraw from the study, giving a final total of 214 cases that met the study criteria and were included (**Figure 1**). Of these 214 cases (epidemiological characteristics are described in **Table 1**), 63.1% (135/214) were male, 72.0% (154/214) had <9 years of education, mean age was 57.5 years (standard deviation 11.1), 58.4% (127/214) had comorbidities, and 10.3% (22/214) had a CCI score ≥3. The most frequent conditions were peripheral vascular disease (33.6%; 72/214) and diabetes (14.5%; 31/214). *H. pylori* infection was positive in 19.2% (41/187) of cases, while 47.2% (101/214) had diffuse Lauren’s subtype tumor, 67.8% (145/214) had non-cardia tumors, 55.6% (119/214) had clinical stage III/IV, and 97.2% (208/214) were treated in the institution (**Table 1**).

**Table 1:** Sociodemographic, lifestyle, and clinical characteristics of 214 patients with gastric adenocarcinoma according to vital status at 1-year follow-up.

	Alive		Deaths		Total		
Variable	n = 173	%	n = 41	%	n = 214	%	p-value
**Sex**							
Male	108	62.4%	27	65.9%	135	63.1%	0.683^1^
Female	65	37.6%	14	34.1%	79	36.9%	
**Age (years)**							
<60	93	53.8%	19	46.3%	112	52.3%	0.393^1^
≥60	80	46.2%	22	53.7%	102	47.7%	
**Marital status**							
Married	130	75.1%	37	90.2%	167	78.0%	0.037^2^
Unmarried	43	24.9%	4	9.8%	47	22.0%	
**Ethnicity**							
White	112	64.7%	26	63.4%	138	64.5%	0.873^1^
Non-white	61	35.3%	15	36.6%	76	35.5%	
**Access to health service**							
Public	41	23.7%	13	31.7%	54	25.2%	0.289^1^
Private or health insurance	132	76.3%	28	68.3%	160	74.8%	
**Education (years)**							
<9 years	42	24.3%	18	43.9%	60	28.0%	0.012^1^
>10 years	131	75.6%	23	56.1%	154	72.0%	
**Tobacco consumption**							
Non-smokers	67	38.7%	19	46.3%	86	40.2%	0.381^1^
Smokers/ex-smokers	107	61.3%	22	53.7%	128	59.8%	
**Alcohol consumption**							
No	86	50.0%	24	58.5%	110	51.6%	0.386^1^
Yes	86	50.0%	17	41.5%	103	48.4%	
Not answered	1	-	-	-	1		
**Body mass index**							
Underweight	9	5.2%	2	4.9%	11	5.1%	0.606^2^
Normal weight	66	38.2%	19	46.3%	85	39.7%	
Overweight	62	35.8%	15	36.6%	77	36.0%	
Obese	36	20.8%	5	12.2%	41	19.2%	
**Comorbidities**							
No	72	41.6%	15	36.6%	87	40.7%	0.470^1^
Yes	101	58.4%	26	63.4%	127	59.3%	
**Charlson Comorbidity Index**							
0 points	72	41.6%	15	36.6%	87	40.7%	0.023^1^
1–2 points	88	50.9%	17	41.5%	105	49.1%	
≥3 points	13	7.5%	9	22.0%	22	10.3%	
**Peripheral vascular disease**							
No	116	67.1%	26	63.4%	142	66.4%	0.658^1^
Yes	57	32.9%	15	36.6%	72	33.6%	
**Diabetes**							
No	153	88.4%	30	73.2%	183	85.5%	0.013^1^
Yes	20	11.6%	11	26.8%	31	14.5%	
**Cerebrovascular disease**							
No	170	98.3%	35	85.4%	205	95.8%	0.002^2^
Yes	3	1.7%	6	14.6%	9	4.2%	
**Myocardial infarction**							
No	162	93.6%	36	87.8%	198	92.5%	0.201^1^
Yes	11	6.4%	5	12.2%	16	7.5%	
***Helicobacter pylori* infection**							
Negative	113	75.3%	33	89.2%	146	68.2%	0.078^2^
Positive	37	24.7%	4	10.8%	41	19.2%	
Not tested	23	-	4	-	27	-	
**Lauren type**							0.181^1^
Intestinal	66	38.2%	10	24.4%	76	35.5%	
Diffuse	80	46.2%	21	51.2%	101	47.2%	
Mixed or NOS adenocarcinoma	27	15.6%	10	24.4%	37	17.3%	
**Histological grade**							
GH1 (well differentiated)	10	7.4%	2	6.5%	12	5.6%	0.410^2^
GH2 (moderately differentiated)	52	38.5%	9	29.0%	61	28.5%	
GH3 (poorly differentiated)	72	53.3%	19	61.3%	91	42.5%	
Undifferentiated	1	0.7%	1	3.2%	2	0.9%	
Information not available	38	-	10	-	48	-	
**Tumor topography**							
Cardia	56	32.4%	13	31.7%	69	32.2%	0.785^1^
Non-cardia	117	67.6%	28	68.3%	145	67.8%	
**HER2**							
Negative	130	89.7%	32	86.5%	162	75.7%	0.304^2^
Doubtful	2	1.4%	2	5.4%	4	1.9%	
Positive	13	9.0%	3	8.1%	16	7.5%	
Not tested	28	-	4	-	32	-	
**Clinical stage**							
I/II	89	51.4%	6	14.6%	95	44.4%	<0.001^1^
III/IV	84	48.6%	35	85.4%	119	55.6%	
**Treatment**							
No	4	2.3%	2	4.9%	6	2.8%	0.324^2^
Yes	169	97.7%	39	95.1%	208	97.2%	

HER2, human epidermal growth factor receptor 2; NOS, not otherwise specified.

^1^ p-Values determined using Pearson’s chi-square test.

^2^ p-Values determined using Fisher’s exact test.

At 1-year follow-up, 41 patients (19.2%) had died (**Figure 1**). Of these deaths, 65.9% (27/41) were male, 43.9% (18/41) had<9 years of education, and 46.3% (19/41) were <60 years. CCI score was ≥3 in 21.9% (9/41) of cases, and the most frequent comorbidities were peripheral vascular disease (36.6%; 15/41) and diabetes (26.8%; 11/41). *H. pylori* infection was positive in 10.8% (4/37) of cases, 65.9% (27/41) had non-cardia tumors, 51.2% (21/41) had the diffuse subtype, 86.5% (32/37) had negative HER2 receptor, 85.4% (35/41) were at clinical stage III/IV, and 95.1% (39/41) received treatment in the institution (**Supplementary Table 1**). Data comparing patients by vital status at 1-year follow-up revealed that deceased patients were more likely to be married (90.2% vs 75.1%, p = 0.037) and had lower education (<9 years; 43.9% vs 24.3%, p = 0.012), more comorbidities (CCI score ≥ 3; 22% vs 7.5%, p = 0.023), diabetes (26.8% vs 11.6%, p = 0.013), cerebrovascular disease (14.6% vs 1.7%, p = 0.002), and clinical stage III/IV (85.4% vs 48.6%, p< 0.001) (**Table 1**).

**Figure 2:**
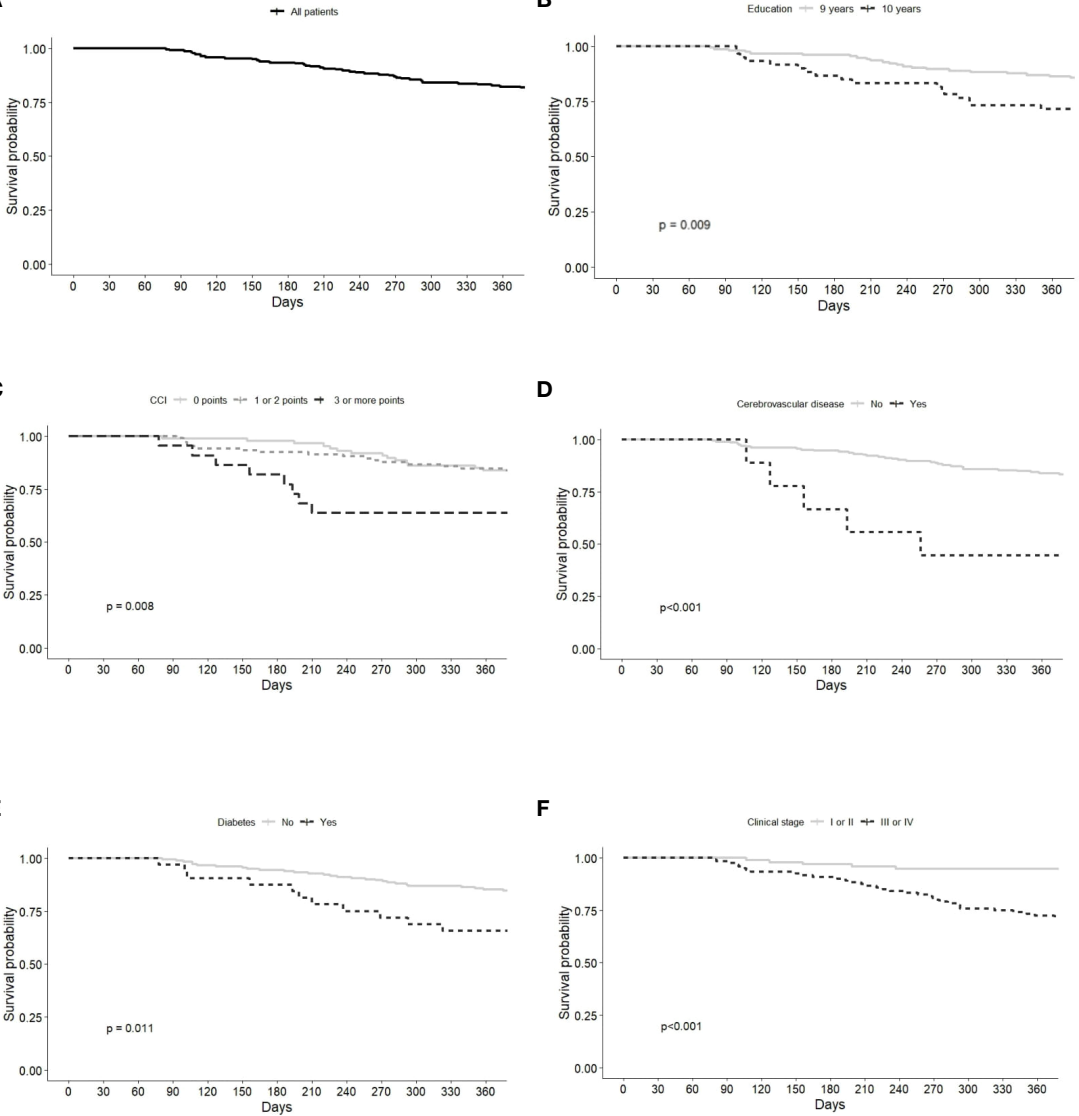
Kaplan-Meier survival curves of Gastric Adenocarcinoma patients grouped by **(A)** all patients, **(B)** education, **(C)** Charlson Comorbidity index (CCI), **(D)** cerebrovascular disease, **(E)** diabetes, **(F)** clinical stage.

Overall survival was 80.8% (95% CI 75.7–86.3%). A significantly lower survival probability was observed in patients with <9 years of education (70.0%), CCI score ≥3 (59.1%), cerebrovascular disease (33.3%), diabetes (64.5%), and clinical stage III/IV (70.6%) (**Figure 2**; **Table 2**).

**Table 2:** One-year overall survival probability of 41 gastric adenocarcinoma patients based on Kaplan–Meier curves with log-rank test.

	Deaths/total	1-year survival	p-Value*
Overall survival	41/214	80.8	-
**Sex**			
Male	27/135	80.0	0.665
Female	14/79	82.3	
**Age (years)**			
<60	19/112	83.0	0.380
≥60	22/102	78.4	
**Marital status**			
Married	37/167	77.8	0.043
Unmarried	4/47	91.5	
**Ethnicity**			
White	26/138	81.2	0.857
Non-white	15/76	80.3	
**Access to health service**			
Public	13/54	75.9	0.293
Private or health insurance	28/160	82.5	
**Education (years)**			
<9 years	18/60	70.0	0.009
>10 years	23/154	85.1	
**Tobacco consumption**			
Non-smokers	19/86	77.9	0.416
Smokers/ex-smokers	22/128	82.8	
**Alcohol consumption**			
No	24/110	78.2	0.302
Yes	17/103	83.5	
Not answered	0/1	-	
**Body mass index**			
Underweight	8/42	81.0	0.379
Normal weight	20/81	75.3	
Overweight	8/50	84.0	
Obese	5/41	87.8	
**Comorbidities**			
No	15/87	82.8	0.478
Yes	26/127	79.5	
**Charlson Comorbidity Index**			
0 points	15/87	82.8	0.008
1–2 points	17/105	83.8	
≥3 points	9/22	59.1	
**Peripheral vascular disease**			
No	26/116	81.7	0.595
Yes	15/72	79.2	
**Diabetes**			
No	30/182	83.5	0.011
Yes	11/32	65.6	
**Cerebrovascular disease**			
No	35/205	82.9	<0.001
Yes	6/9	33.3	
**Myocardial infarction**			
No	36/198	81.8	0.123
Yes	5/16	68.8	
***Helicobacter pylori* infection**			
Negative	33/146	77.4	0.083
Positive	4/41	90.2	
Not tested	4/27	-	
**Lauren type**			
Intestinal	10/76	86.8	0.201
Diffuse	21/100	79.0	
Mixed or NOS adenocarcinoma	10/38	73.7	
**Histological grade**			
GH1 (well differentiated)	2/12	83.3	0.680
GH2 (moderately differentiated)	9/61	85.2	
GH3 (poorly differentiated)	19/91	79.1	
Undifferentiated	1/2	50.0	
Information not available	10/48	-	
**Tumor topography**			
Cardia	14/77	81.8	0.780
Non-cardia	27/137	80.3	
**HER2**			
Negative	31/162	80.7	0.113
Doubtful	2/4	50.0	
Positive	3/16	81.3	
Not tested	5/32	-	
**Clinical stage**			
I/II	6/95	93.7	<0.001
III/IV	35/119	70.6	
**Treatment**			
No	2/6	66.7	0.482
Yes	39/208	81.3	

HER2, human epidermal growth factor receptor 2; NOS, not otherwise specified.

*Log-rank test.

**Figure 3:**
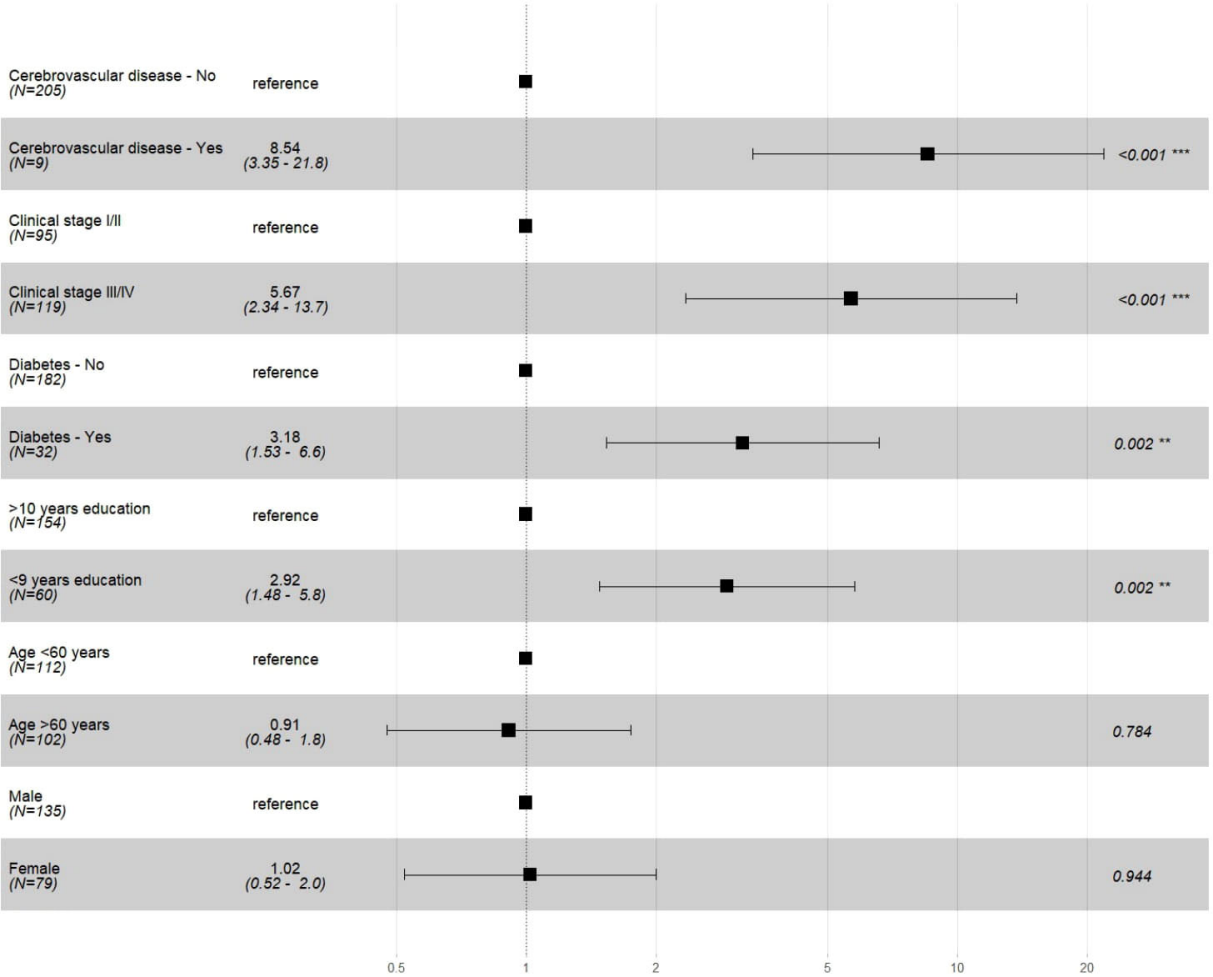
Multivariable Cox regression analysis of selected prognostic factors in Gastric Adenocarcinoma cases. * Schoenfeld risk proportionality test of the multiple model p=0.990; model adjusted for variables age and sex.

Cox univariate regression analyses showed that the risk of death clearly increased in groups with lower education, CCI score ≥3, cerebrovascular disease, diabetes, and clinical stage III/IV. After age and sex were adjusted in the multivariable model, prognostic factors were cerebrovascular disease (HR 8.54, 95% CI 3.35–21.8), stage III/IV (HR 5.67, 95% CI 2.34–13.72), diabetes (HR 3.18, 95% CI 1.53–6.6), and <9 years of education (HR 2.92, 95% CI 1.48–5.8) (**Figure 3**; **Table 3**).

**Table 3:** Cox univariate regression analyses of 1-year overall survival prognostic factors of 41 patients with gastric adenocarcinoma.

Variable	Category	Univariate HR (95% CI)	p-value
**Sex**	Female	1	
	Male	1.15 (0.60–2.20)	0.666
**Age (years)**	<60	1	
	≥60	1.31 (0.71–2.43)	0.382
**Marital status**	Married	1	
	Unmarried	1.19 (0.76–1.87)	0.447
**Ethnicity**	Non-White	1	
	White	0.94 (0.5–1.78)	0.857
**Access to health service**	Public	1	
	Private or health insurance	1.42 (0.77–2.74)	0.296
**Education (years)**	>10 years	1	
	<9 years	2.21 (1.19–4.10)	0.012
**Tobacco consumption**	Non-smokers	1	
	Smokers/ex-smokers	0.78 (0.42–1.43)	0.418
**Alcohol consumption**	No	1	
	Yes	0.72 (0.39–1.34)	0.304
**Body mass index**	Underweight	1	
	Normal weight	1.14 (0.27–4.91)	0.856
	Overweight	0.98 (0.22–4.29)	0.979
	Obese	0.59 (0.11–3.05)	0.530
**Comorbidities**	No	1	
	Yes	1.26 (0.67–2.37)	0.479
**Charlson Comorbidity Index**	0 points	1	
	1–2 points	0.96 (0.48–1.93)	0.914
	≥3 points	3.00 (1.31–6.86)	0.009
**Peripheral vascular disease**	No	1	
	Yes	1.19 (0.63–2.24)	0.596
**Myocardial infarction**	No	1	
	Yes	2.05 (0.81–5.24)	0.132
**Cerebrovascular disease**	No	1	
	Yes	5.76 (2.41–13.76)	<0.001
**Diabetes**	No	1	
	Yes	2.39 (1.20–4.78)	0.009
***Helicobacter pylori* infection**	Negative	1	
	Positive	0.41 (0.14–1.16)	0.094
**Lauren type**	Diffuse	1	
	Intestinal	0.61 (0.29–1.29)	0.197
	Mixed or NOS adenocarcinoma	1.32 (0.62–2.80)	0.473
**Histological grade**	GH1 (well differentiated)	1	
	GH2 (moderately differentiated)	0.91 (0.20–4.19)	0.900
	GH3 (poorly differentiated)	1.23 (0.30–5.49)	0.740
	Undifferentiated	4.19 (0.38–46.35)	0.242
**Tumor topography**	Cardia	1	
	Non-cardia	1.10 (0.57–2.09)	0.780
**HER2**	Negative	1	
	Doubtful	3.96 (0.95–16.54)	0.059
	Positive	0.97 (0.30–3.16)	0.958
**Clinical stage**	I/II	1	
	III/IV	5.27 (2.22–12.53)	<0.001
**Treatment**	No	1	
	Yes	0.60 (0.15–2.50)	0.487

HER2, human epidermal growth factor receptor 2; HR, hazard ratio; CI, confidence interval; NOS, not otherwise specified.

**Figure 4:**
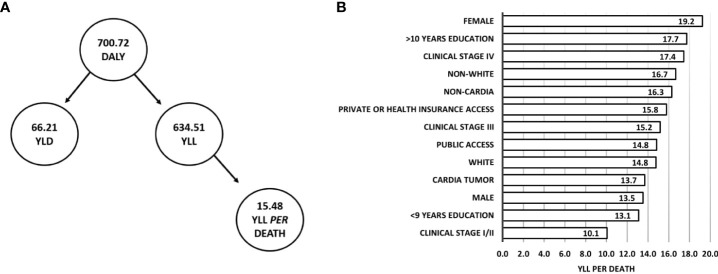
DALY (YLD plus YLL) and YLL per death at 1-year follow-up of Gastric Adenocarcinoma. **(A)** Flowchart of DALY composition and average YLL per death. **(B)** YLL per death according to clinical and sociodemographic characteristics.

Of the 214 participants in the study, at 1-year follow-up, there was 700.72 DALY, of which 634.51 (90.55%) corresponded to YLL and 66.21 (9.45%) to YLD. An average of 15.48 years was lost per death, and YLL per death was higher in individuals who were female (19.24 YLL per death), had >10 years of education (18.99 YLL per death), had clinical stage IV (17.44 YLL per death), and were non-white (16.68 YLL per death) (**Figure 4**).

Gender comparison showed that clinical stage III/IV was more frequent in women (92.9% vs 81.5%), whereas alcohol consumption (55.6% vs 14.3%) and tobacco consumption (70.4% vs 21.4%) were more frequent in men (**Table 4**).

**Table 4:** Sociodemographic, lifestyle, and clinical characteristics of 41 patients with gastric adenocarcinoma who died in the first year of follow-up, by sex.

	Male		Female		Total		
	n = 27	%	n = 14	%	n = 41	%	p-value
**Age (years)**							
<60	11	40.7%	8	57.1%	19	46.3%	0.318^1^
≥60	16	59.3%	6	42.9%	22	53.7%	
**Marital status**							
Married	3	11.1%	1	7.1%	4	9.8%	1.000^2^
Unmarried	24	88.9%	13	92.9%	37	90.2%	
**Ethnicity**							
White	18	66.7%	8	57.1%	26	63.4%	0.548^1^
Non-white	9	33.3%	6	42.9%	15	36.6%	
**Access to health service**							
Public	9	33.3%	4	28.6%	13	31.7%	1.000^2^
Private or health insurance	18	66.7%	10	71.4%	28	68.3%	
**Education (years)**							
<9 years	11	40.7%	7	50.0%	18	43.9%	0.571^1^
>10 years	16	59.3%	7	50.0%	23	56.1%	
**Tobacco consumption**							
Non-smokers	8	29.6%	11	78.6%	19	46.3%	0.007^2^
Smokers/ex-smokers	19	70.4%	3	21.4%	22	53.7%	
**Alcohol consumption**							
No	12	44.4%	12	85.7%	24	58.5%	0.018^2^
Yes	15	55.6%	2	14.3%	17	41.5%	
**Body mass index**							
Underweight	1	3.7%	1	7.1%	2	4.9%	0.538^2^
Normal weight	11	40.7%	8	57.1%	19	46.3%	
Overweight	12	44.5%	3	21.4%	15	36.6%	
Obese	3	11.1%	2	14.4%	5	12.2%	
**Comorbidities**							
No	10	37.0%	5	35.7%	15	36.6%	0.934^1^
Yes	17	63.0%	9	64.3%	26	63.4%	
**Charlson Comorbidity Index**							
0 points	10	37.0%	5	35.7%	15	36.6%	0.184^2^
1–2 points	9	33.3%	8	57.1%	17	41.5%	
≥3 points	8	29.6%	1	7.1%	9	22.0%	
**Peripheral vascular disease**							
No	17	63.0%	9	64.3%	26	63.4%	0.934^1^
Yes	10	37.0%	5	35.7%	15	36.6	
**Diabetes**							
No	19	70.4%	11	78.6%	30	73.2%	0.719^2^
Yes	8	29.6%	3	21.4%	11	26.8%	
**Cerebrovascular disease**							
No	23	85.2%	12	85.7%	35	85.4%	1.000^2^
Yes	4	14.8%	2	14.3%	6	14.6%	
**Myocardial infarction**							
No	23	85.2%	13	92.9%	36	87.8%	0.645^2^
Yes	4	14.8%	1	7.1%	5	12.2%	
***Helicobacter pylori* infection**							
Negative	23	95.8%	10	76.9%	33	89.2%	0.077^2^
Positive	1	4.2%	3	23.1%	4	10.8%	
Not tested	3	-	1	-	4	-	
**Lauren classification**							
Intestinal	8	29.6%	2	14.3%	10	24.4%	0.433^2^
Diffuse	12	44.4%	9	64.3%	21	51.2%	
Mixed or NOS adenocarcinoma	7	25.9%	3	21.4%	10	24.4%	
**Histological grade**							
GH1 (well differentiated)	2	8.7%	0	0.0%	2	6.5%	
GH2 (moderately differentiated)	7	30.4%	2	25.0%	9	29.0%	
GH3 (poorly differentiated)	13	56.5%	6	75.0%	19	61.3%	0.538^2^
Undifferentiated	1	4.3%	0	0.0%	1	3.2%	
Information not available	4	-	6	-	10	-	
**Tumor topography**							
Cardia	11	40.7%	3	21.4%	14	34.1%	0.305^2^
Non-cardia	16	59.3%	11	78.6%	27	65.9%	
**HER2**							
Negative	21	87.5%	11	84.6%	32	86.5%	0.900^2^
Doubtful	1	4.2%	1	7.7%	2	5.4%	
Positive	2	8.3%	1	7.7%	3	8.1%	
Not tested	3	-	1	-	4	-	
**Clinical stage**							
I/II	5	18.5%	1	7.1%	6	14.6%	0.645^2^
III/IV	22	81.5%	13	92.9%	35	85.4%	
**Treatment**							
No	1	3.7%	1	7.1%	2	4.9%	0.572^2^
Yes	26	96.3%	13	92.9%	39	95.1%	

HER2, human epidermal growth factor receptor 2; NOS, not otherwise specified.

^1^ p-Values determined using Pearson’s chi-square test.

^2^ p-Values determined using Fisher’s exact test.

## Discussion

This study reported the status of gastric adenocarcinoma patients at 1-year follow-up for overall survival probability and prognostic factors, DALY, YLL, YLD, and YLL per death. Overall survival was 80.8% (95% CI 75.7%–86.3%). Survival probability was lower in patients with <9 years of education, CCI score ≥3, cerebrovascular disease, diabetes, and clinical stage III/IV. In the first year post-diagnosis, there was 700.72 DALY, of which 90.55% was YLL, an average of 15.48 years was lost per death, and women had the highest YLL per death.

This study showed that 1-year overall survival was higher compared to that described by Poonyam et al. (2021) in Thailand (36) and that of Carneseca et al. (2013) in another study in Brazil (37). However, the overall survival was comparable to rates reported in Holland and Portugal (71% and 82%, respectively) (38, 39). The contrasting results of the present study could be explained by advances in cancer treatment (40) and the specific sociodemographic profile of the population investigated. In a representative sample of the Brazilian population, 56.2% self-declared as non-white, 51.1% had an educational level of elementary or lower (<9 years of education), and only one-third (28.5%) had private health insurance (23, 41). By contrast, in the present cohort, patients were predominantly of white ethnicity, had >10 years of education, and had access to the healthcare service by private or health insurance, differing from the national average.

Patients with lower educational levels tend to have lower survival rates (4) and, consequently, poorer prognosis (42). Education is also associated with socioeconomic level, eating habits, sanitary conditions (3), and access to diagnosis and treatment (43). Lower educational level was also associated with lower access to private or health insurance, lower survival rate, and poorer prognosis in the current study; socioeconomic factors have an impact on the prognosis of gastric adenocarcinoma.

The clinical stage is typically associated with a poorer prognosis (13–15, 44–46). For instance, in this study, stage III/IV gastric adenocarcinoma cases had an increased risk of death as compared to stage I/II patients, and lower overall survival. Similarly, a study performed in northern Brazil reported 86.4% overall survival in patients with early-stage cancer in the first year and 44.6% overall survival in advanced cases (47). Moreover, studies carried out in São Paulo by Ramos et al. (2018) and Costa et al. (2015) recorded 87.3% overall survival for stage I and 27.2% overall survival for stage IV (21) in the first year. Furthermore, after neoadjuvant therapy, 95.4% and 78.9% overall survival were recorded for stages I/II and III/IV (48), respectively. Comorbidities were also associated with an increased risk of death in patients with gastric cancer (10), and a CCI score ≥3 was associated with lower overall survival, supporting previous studies (11, 49). This result confirmed that comorbidities exist at gastric adenocarcinoma diagnosis and that the type and severity of the comorbidity are important for therapeutic planning. In our study, diabetes and cerebrovascular disease impacted prognosis. According to Shimada et al. (2020), patients who died by the first year of follow-up had a higher rate of cerebrovascular diseases before cancer diagnosis (49). This relationship was corroborated by the findings of the current study. Although cerebrovascular diseases are associated with a poorer prognosis in gastric adenocarcinoma cases, studies investigating the impact of cerebrovascular diseases are scarce. Previous studies on sarcoma, colorectal, and breast cancers found an association between comorbidities and therapeutic indication, which could affect survival (50–53). The role of diabetes in gastric cancer risk and prognosis is uncertain; however, some studies suggested that diabetes promotes cell growth, proliferation, and chemoresistance of gastric adenocarcinoma (54, 55). A large cohort study by Dabo et al. (2021) concluded that, while diabetes is associated with cardia gastric cancer, it is not related to gastric cancer overall (56). In the current study, diabetes before gastric adenocarcinoma was correlated with lower overall survival probability, higher risk of death, and, consequently, poorer prognosis, supporting existing epidemiological studies (57–60).

In 2016, approximately 17.9 million years of life was lost due to gastric cancer worldwide, of which 98% was due to YLL (5). This rate was slightly higher than that obtained in our study, in which approximately 90% of DALY corresponded to a total YLL of 634.51 years. These data might reflect differences in access to treatment at a referral cancer center and completeness of follow-up. Although men between the ages of 30 and 70 are more likely to develop gastric cancer (one in 32 men vs one in 81 women) (5), women in this cohort had higher YLL per death. Kim et al. (2016) found an association between gastric adenocarcinoma diagnosis in young women and poorer prognosis (59). In this cohort, female gender was not a prognostic factor; however, the exploratory analysis revealed that most women were diagnosed at stage III/IV and were younger than 60, explaining the higher YLL per death in women. Similar results were obtained in population-based studies conducted in the United States (11.6 YLL and 12.5 YLL per death for men and women, respectively) (61) and Japan (12.0 YLL and 13.5 YLL per death for men and women, respectively) (62). The hospital-based findings for the cohort of the current study reflected the population profile.

The findings of the current study were consistent with other population-based studies. However, the small sample size and the number of events (deaths) could have reduced the power and widened the confidence interval. Furthermore, the current study was conducted at a single center, limiting population-level inferences. Given the low number of events, if an interaction between any of the variables was present, it was a natural phenomenon whose likelihood is unaffected by our sample size.

At a single cancer center, 1-year overall survival probability was approximately 80% in patients with gastric adenocarcinoma. Patients with a higher risk of death had cerebrovascular disease, advanced clinical staging, diabetes, and/or lower educational level. Approximately 700 years of DALY was documented, with women having the highest YLL per death. Because this study was conducted at a single cancer center, the results might not be representative of the general population. To the best of our knowledge, this study was the first to assess gastric adenocarcinoma DALY, YLL, and YLL per death at the first year of follow-up in a hospital cohort in Brazil.

## Data availability statement

The raw data supporting the conclusions of this article will be made available by the authors, without undue reservation.

## Ethics statement

The studies involving human participants were reviewed and approved by Ethics and Research Committee of the AC Camargo Cancer Center under protocol n°2169/165. The patients/participants provided their written informed consent to participate in this study.

## Author contributions

All authors participated in this study. TT, GF, and MC, concepts and design. TT, GF, and MC, data acquisition. TT, GF, and MC, data analysis and interpretation. GF and MC, study supervision. TT, GF, and MC, writing, review, and revision of manuscripts. All authors contributed to the article and approved the submitted version.

## Funding

The project “Epidemiology and Genomics of Gastric Adenocarcinomas in Brazil” was funded by the Fundação de Amparo à Pesquisa do Estado de São Paulo-FAPESP under grant 2014/26897-0. The present study received financial support from the Coordenação de Aperfeiçoamento de Pessoal de Nível Superior—Brasil (CAPES)—Finance Code 001.

## Acknowledgments

We thank the staff of the Group of Epidemiology and Statistics on Cancer, A. C. Camargo Cancer Center, São Paulo, Brazil, for supporting the study.

## Conflict of interest

The authors declare that the study was conducted in the absence of any commercial or financial relationships that could be construed as a potential conflict of interest.

## Publisher’s note

All claims expressed in this article are solely those of the authors and do not necessarily represent those of their affiliated organizations, or those of the publisher, the editors and the reviewers. Any product that may be evaluated in this article, or claim that may be made by its manufacturer, is not guaranteed or endorsed by the publisher.
