# Supplementary material for: Gastric adenocarcinoma: 1-year overall survival, disability-adjusted life years, years of life lost, and prognostic factors—a single-institution experience
Source: Front Oncol. 2022 Sep 8;12:918833. doi: 10.3389/fonc.2022.918833 (PMC9493081; doi:10.3389/fonc.2022.918833)
Supplement: Supplementary file 1 [file Table_1.docx]

Supplementary table 1: Treatment characteristics of 41 cases with Gastric Adenocarcinoma who died in the first year follow-up.

| Case | Tratment | Treatment information |
| --- | --- | --- |
| 1 | none | - |
| 2 | none | - |
| 3 | Surgery | Subtotal gastrectomy + Lymph node dissection D2/ retroperitoneal |
| 4 | Surgery | Subtotal gastrectomy + Lymph node dissection D1 |
| 5 | Surgery | Subtotal gastrectomy + Lymph node dissection D1 |
| 6 | Chemotherapy + radiotherapy | Carboplatin + Paclitaxel (4 cycles) |
| 7 | Chemotherapy | EOX + EOF (3 cycles) |
| 8 | Chemotherapy | FOLFOX + XELOX (6 cycles)  Carboplatin + Paclitaxel (3 cycles) |
| 9 | Chemotherapy | CF + XP (3 cycles) |
| 10 | Chemotherapy | DCF (8 cycles)  FOLFOX + XELOX (2 cycles)  FOLFIRI (1 cycles) |
| 11 | Chemotherapy | FOLFOX + XELOX (6 cycles) |
| 12 | Chemotherapy + radiotherapy | FOLFOX + XELOX (16 cycles)  FOLFIRI (4 cycles)  Irinotecan (1 cycle) |
| 13 | Chemotherapy | FLOT (14 cycles)  Irinotecan (2 cycles) |
| 14 | Chemotherapy | FOLFOX + XELOX (2 cycles) |
| 15 | Chemotherapy | FOLFOX + XELOX (10 cycles)  FOLFIRI (3 cycles)  Paclitaxel (1 ciclo) |
| 16 | Chemotherapy | FOLFOX + XELOX (2 cycles) |
| 17 | Chemotherapy | FOLFOX + XELOX (4 cycles) |
| 18 | Chemotherapy | FOLFOX + XELOX (7 cycles)  FOLFIRI (8 cycles) |
| 19 | Chemotherapy | FLOT (11 cycles)  Irinotecan (1 ciclo) |
| 20 | Chemotherapy | FOLFOX (5 cycles)  Irinotecan (5 cycles)  Paclitaxel (1 cycle) |
| 21 | Chemotherapy | Cisplatin + Docetaxel (4 cycles)  Irinotecan (1 cycle) |
| 22 | Chemotherapy | FOLFOX + Trastuzumab (7 cycles)  Paclitaxel (1 cycle) |
| 23 | Chemotherapy | FOLFOX + XELOX (9 cycles)  Paclitaxel (1 cycle) |
| 24 | Chemotherapy | FLOT (8 cycles)  FOLFIRI (1 cycle) |
| 25 | Chemotherapy | FOLFOX + XELOX (1 cycle) |
| 26 | Chemotherapy | FOLFOX + XELOX (4 cycles) |
| 27 | Chemotherapy | FOLFOX + XELOX (4 cycles)  Paclitaxel + Ramucirumab (2 cycles) |
| 28 | Chemotherapy | FOLFOX + Trastuzumab (17 cycles) |
| 29 | Chemotherapy | FOLFOX + XELOX (14 cycles) |
| 30 | Chemotherapy | FOLFOX + XELOX (6 cycles)  FOLFIRI (2 cycles) |
| 31 | Chemotherapy | FOLFOX + XELOX (13 ciclo)  Paclitaxel (1 cycles) |
| 32 | Chemotherapy | FOLFOX + XELOX (4 cycles) |
| 33 | Chemotherapy    Surgery | FLOT (4 cycles)  FOLFOX (4 cycles)  Irinotecan (1 ciclo)  Total gastrectomy + Lymph node dissection D2 |
| 34 | Chemotherapy  Surgery | FOLFOX + XELOX (4 cycles)  Total gastrectomy + Lymph node dissection D2 |
| 35 | Chemotherapy  Surgery | FOLFOX + XELOX (8 cycles)  Total gastrectomy + Lymph node dissection D2/ retroperitoneal |
| 36 | Chemotherapy  Surgery | CF + XP (5 cycles)  Subtotal gastrectomy + Lymph node dissection D2 |
| 37 | Chemotherapy  Surgery | FOLFOX + XELOX (6 cycles)  Total gastrectomy + Lymph node dissection D2 |
| 38 | Chemotherapy  Surgery | FLOT (6 cycles)  FOLFIRI (2 cycles)  Subtotal gastrectomy + Lymph node dissection D2/ retroperitoneal |
| 39 | Chemotherapy  Surgery | FLOT (8 cycles)  Total gastrectomy + Lymph node dissection D2 |
| 40 | Chemotherapy  Surgery | FOLFOX + XELOX (5 cycles)  Subtotal gastrectomy + Lymph node dissection D1 |
| 41 | Chemotherapy  Surgery | FOLFOX + XELOX (3 cycles)  Subtotal gastrectomy + Lymph node dissection D2 |

CF: Cisplatin and fluorouracil; DCF: docetaxel, cisplatin and fluorouracil; EOF: epirubicin, oxaliplatin and fluorouracil; EOX: epirubicin, oxaliplatin and capecitabine; FLOT: fluorouracil, leucovorin, oxaliplatin and docetaxel; FOLFIRI: fluorouracil, leucovorin and irinotecan; FOLFOX: fluorouracil, leucovorin and oxaliplatin; XELOX: oxaliplatin and capecitabine; XP: capecitabine and cisplatin
